# Recent Update on Applications of Quaternary Ammonium Silane as an Antibacterial Biomaterial: A Novel Drug Delivery Approach in Dentistry

**DOI:** 10.3389/fmicb.2022.927282

**Published:** 2022-09-23

**Authors:** Ranjeet Ajit Bapat, Abhishek Parolia, Tanay Chaubal, Ho Jan Yang, Prashant Kesharwani, Khoo Suan Phaik, Seow Liang Lin, Umer Daood

**Affiliations:** ^1^Restorative Dentistry Division, School of Dentistry, International Medical University, Kuala Lumpur, Malaysia; ^2^School of Pharmaceutical Education and Research, Jamia Hamdard (Hamdard University), New Delhi, India; ^3^Division of Clinical Oral Health, School of Dentistry, International Medical University, Kuala Lumpur, Malaysia

**Keywords:** quaternary ammonium silane, Sortase-A, antimicrobial, antibacterial, biomaterial, dentistry, drug delivery, irrigant

## Abstract

Quaternary ammonium silane [(QAS), codename – k21] is a novel biomaterial developed by sol-gel process having broad spectrum antimicrobial activities with low cytotoxicity. It has been used in various concentrations with maximum antimicrobial efficacy and biocompatibility. The antimicrobial mechanism is displayed *via* contact killing, causing conformational changes within the bacterial cell membrane, inhibiting Sortase-A enzyme, and causing cell disturbances due to osmotic changes. The compound can attach to S1' pockets on matrix metalloproteinases (MMPs), leading to massive MMP enzyme inhibition, making it one of the most potent protease inhibitors. Quaternary ammonium silane has been synthesized and used in dentistry to eliminate the biofilm from dental tissues. QAS has been tested for its antibacterial activity as a cavity disinfectant, endodontic irrigant, restorative and root canal medication, and a nanocarrier for drug delivery approaches. The review is first of its kind that aims to discuss applications of QAS as a novel antibacterial biomaterial for dental applications along with discussions on its cytotoxic effects and future prospects in dentistry.

## Introduction

Dental caries and periodontal disease are two of the most common diseases affecting the oral cavity. Based on the Global Burden of Disease in 2017, dental caries in permanent teeth had a prevalence of 29.4%. For severe periodontitis, the age-standardized prevalence was 9.8%, with 796 million prevalent cases (Bernabe et al., 2020). The pathogenesis of these two diseases is intricately linked with the oral microbiomes within the dental plaque, a polymicrobial biofilm characterized by diverse communities of microorganism embedded within an extracellular matrix and associated with interfaces (Davey and O'toole, 2000). Following frequent consumption of fermentable carbohydrates, acidogenic/aciduric organisms produce acidic by-products that lead to progressive demineralization and loss of calcium and phosphate ions from the tooth structure. These caries-associated bacteria include, but are not limited to, *Streptococcus mutans* (*S. mutans*), *non-mutans Streptococci, Lactobacillus, Bifidobacterium*, and *Actinomyces* (Takahashi and Nyvad, 2011). If left untreated, the carious lesion will extend deep to involve the pulpal space, causing root canal infection that will manifest as pulpitis or apical periodontitis (Nair, 2004).

The microbial profile of a primary root canal infection is dominated by *Fusobacterium, Porphyromonas, Prevotella, Parvimonas, Tannerella, Treponema, Dialister, Filifactor, Actinomyces, Olsenella, and Pseudoramibacter* (Siqueira and Rôças, 2009). In cases of endodontic failure with resultant persistent or secondary root canal infection, there is a preponderance of facultative anaerobes and gram-positive bacteria inside the root canals (Gomes and Herrera, 2018). A quintessential bacterium from this group is *Enterococcus faecalis (E. faecalis)*, a facultative gram-positive bacterium capable of surviving in harsh environment and invading the dentinal tubules (Love, 2001). Besides physical disruption of the plaque biofilm *via* procedures such as professional plaque removal and root canal instrumentation, the bacteria load can also be reduced by adjunctive chemical means, such as the use of antiseptic agent. One of the most widely used antiseptics in dentistry today is chlorhexidine (CHX) digluconate. Some evidence exists that supports CHX use for plaque control, gingivitis, and symptomatic management of certain viral infections. However, CHX use is associated with some adverse effects, such as teeth staining, emerging antimicrobial resistance, and rare anaphylaxis. The 2% CHX concentration application may cause oversaturation on the dentine substrate, leading to a rapid release of CHX excess (Carrilho et al., 2010). CHX preparations also delay the time for recontamination of coronally sealed canals. However, the time taken for microorganisms into the root canal was more with CHX solutions as compared to that of other disinfectant material (calcium hydroxide). Moreover, its effectiveness in the prevention and management of dental caries, chronic periodontitis, and peri-implantitis is less clear (Brookes et al., 2020).

Other antiseptics for dental application include sodium hypochlorite (NaOCl), hydrogen peroxide, citric acid, EDTA, povidone iodine, phenols, and essential oils. Each agent has its own advantages and disadvantages, and is indicated for different, sometimes overlapping clinical situations. The authors of a recent systematic review that compared the antibacterial efficacy between CHX and NaOCl in root canal disinfection suggested that there is a need for the development of a more potent antibacterial agent, because neither can completely eliminate the bacterial population from the root canal, and the interaction between them yields a precipitate that can occlude dentinal tubules (Ruksakiet et al., 2020). Insufficient elimination of microorganisms and their by-products is observed *in vitro* with gold standard irrigants such as NaOCl (Kenawy et al., 2007). Even though NaOCl has an excellent pulp-dissolving property with a distinct antibacterial spectrum (Isquith et al., 1972), it is cytotoxic to the periapical tissues (Battice and Hales, 2016) and cannot remove the smear layer completely (Makvandi et al., 2018). Research has illustrated modifications in dentin composition after use of NaOCl as an irrigant in root canal therapy (Tartari et al., 2016).

Due to the limitations of conventional irrigant solutions, irrigants with nanoparticles have been developed. Among the use of organic nanoparticles, irrigants with chitosan nanoparticles have depicted enhanced antibacterial effect by emission of singlet oxygen species. These are suggested to be used as finishing rinses as root canal irrigants for an efficient outcome (Shrestha and Kishen, 2014). As NaOCl can cause hemolysis and soft tissue ulceration if extruded periapically by amalgamation of graphene into silver nanoparticles, toxic effects on soft tissue and bone are reduced (Hu et al., 2011). Poly (lactic) co-glycolic acid nanoparticles combined with photoactive drugs are utilized as a vital aid in the elimination of bacteria from endodontic canals (Raura et al., 2020). Among the non-organic nanoparticles, it is observed that the use of bioactive glass-based nanoparticles having increased pH concentration and consistent flow of alkaline material is very effective for root canal disinfection (Waltimo et al., 2009). Among metallic nanoparticles, the nano-silver solution possessing particle size of 1–2 nm showed its capability in penetration of dentinal surfaces and generated a film casing the dentin surface of the root canal prior sealer application (Razumova et al., 2022). Metallic oxide nanoparticles like magnesium oxide nanoparticles exhibited long-lasting efficiency in elimination of *E. faecalis* superior to the NaOCl irrigant (Kishen et al., 2008). As root canal infections are polymicrobial, tetracycline topical applications have been used for local application as antibacterial agents. Mostly the antibiotics are used as intracanal medicaments in paste forms is various combinations and not as intracanal irrigant solutions. EDTA irrigation can be beneficial in disruption of biofilm (Segura-Egea et al., 2017). Few mixtures such as tetraclean (doxycycline, citric acid, and a surfactant), BioPure MTAD (mixture of doxycycline, citric acid, and a detergent) and QMix having an antimicrobial, a chelating agent, and one surfactant have also been used for final irrigation, although evidence is still lacking in relation to its biocompatibility (Boutsioukis and Arias-Moliz, 2022). Superoxide water and ozonated water have also been used for disinfection of root canal systems (Solovyeva and Dummer, 2000; Huth et al., 2009), although long-term results are needed to be used on a consistent basis for root canal disinfection.

Given the limitations of existing antiseptics and the fact that an ideal antiseptic agent does not exist currently, there remains the need to look for novel antimicrobial biocides that can further lead to improvement in clinical outcomes during dental practice. Antimicrobial polymers represent a class of biocides that are increasingly being utilized in the industrial, healthcare, and consumer end-use sectors. It is hoped that the use of antimicrobial polymers can overcome the drawbacks associated with low-molecular weight antimicrobial agents, chiefly environmental toxicity, and short-term activity (Kenawy et al., 2007). Biocide releasing polymers, such as those incorporating silver nanoparticles or antibiotics, can rapidly eliminate microbes, but the subsequent depletion of the leachable biocides can reach a sub-inhibitory level that could potentially promote antimicrobial resistance. It is apparent that this group of biocides lacks a sustained antimicrobial activity. To circumvent this limitation, a class of contact-killing biocidal polymer is conceived. This group of polymers is predicated on the covalent immobilization of antimicrobial functional groups on the material surface. This type of biocide is known as quaternary ammonium compounds (QAC). QAC are by-products of ammonium compounds, with all four of the hydrogen bonded to nitrogen are replaced by hydrocarbyl groups. The overall formulas are R4NX, four R, hydrocarbyl, which may be similar or different; furthermore, X is a halogen anion in utmost cases and can also be acid radical, like RCOO, HSO4, RCOO, etc. Quaternary ammonium compounds (QACs) have been consistently used since 1930s, with no apparent decrease in their effectiveness (Gilbert and Moore, 2005). In the 1970s, QAC were first combined into mouthwashes to impede oral biofilms containing two quaternary ammonium compounds (Rosa and Sturzenberger, 1967; Bonesvoll and Gjermo, 1978). In 1994, for the first time, QAC-based antimicrobial monomer was developed by combining QAC along with methyl acryloyl groups. The structure was comparable to cetylpyridinium chloride (CPC), which has been used in toothpaste and mouthwashes as antibacterial biomaterial. Thus, research has reported that combining QAC and methacryloyl groups, antibacterial quaternary ammonium antimicrobial monomer comprising different alkyl chain lengths is established (Imazato et al., 1994). Since then, diverse kinds of quaternary ammonium monomers have been developed and combined into composite adhesive systems, glass ionomer cement (GIC), and resins to attain an antibacterial outcome. With evolution of science, research scientists are exploring various alterations in the structure of QAC to improve their performance with maximum biocompatibility (Zhang et al., 2018). One such example is the quaternary ammonium silane (QAS), which is able to attach covalently to the substrate surfaces *via* Si-O linkages by virtue of their reactive silanol groups (Isquith et al., 1972). QAS was reported as early as 1970s, with Dow Corning corporation registering the 3-(trimethoxysilyl)-propyldimethyloctadecyl ammonium chloride (SiQAC) with the US Environmental Protection Agency (Battice and Hales, 2016). A quaternary ammonium silane (QAS; codenamed K21; C92H204Cl4N4O12Si5; CAS No. 1566577-36-3) is produced by the sol–gel method, with a reaction of 3- (triethoxysilyl)-propyldimethyloctadecyl ammonium chloride with tetraethoxysilane (TEOS) as the anchoring unit, utilizing a molar ratio of 4:1 (Rossi-Fedele et al., 2011; Daood et al., 2020b). The usage of TEOS as an anchoring unit facilitates a three-dimensional organically modified silicate network produced by condensation of more tetra- and triethoxysilane molecules with remaining silanol groups within the molecule. The by-product of this sol–gel reaction is ethanol instead of methanol, which has been found to be toxic for oral use (Tiller et al., 2001).

**Figure 1:**
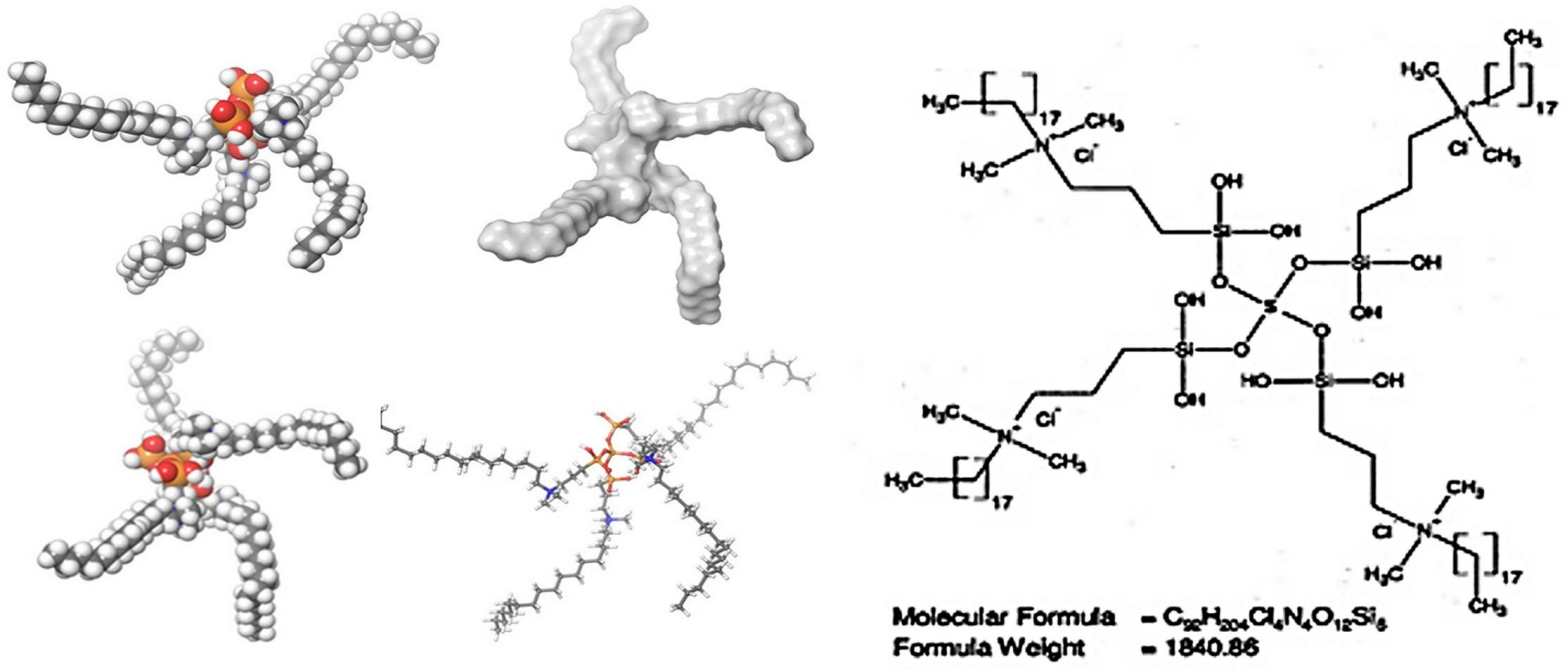
Chemical and molecular structure of QAS.

The structure of quaternary ammonium compounds (QAC) contains a positively charged nitrogen atom (hydrophobic head) having four bonds to the alkyl hydrophilic tail (Makvandi et al., 2018). One of the constituents is a long lipophilic chain containing carbon atoms [no of carbon atoms (C) varies from C8 to C18] (see Figure 1). In cases of bis-QAC and multi-QACs, the spacer links the head part to the tail (Vereshchagin et al., 2021). QACs are normally stable and water soluble. The quaternary amine that is positively charged gets attracted toward negatively charged cell membrane of microorganisms disrupting the membrane and leading to leakage of cytoplasmic constituents (Namba et al., 2009). The long alkyl chain offers a hydrophobic piece corresponding to the bilayer of the cell wall of organisms. Thus, this review highlights antibacterial action, various dental applications, information on cytotoxicity of QAS to be considered as a novel biomaterial that can be routinely used for clinical applications in dentistry.

## Mechanism of Actions of QAS

### Antibacterial Action

**Figure 2:**
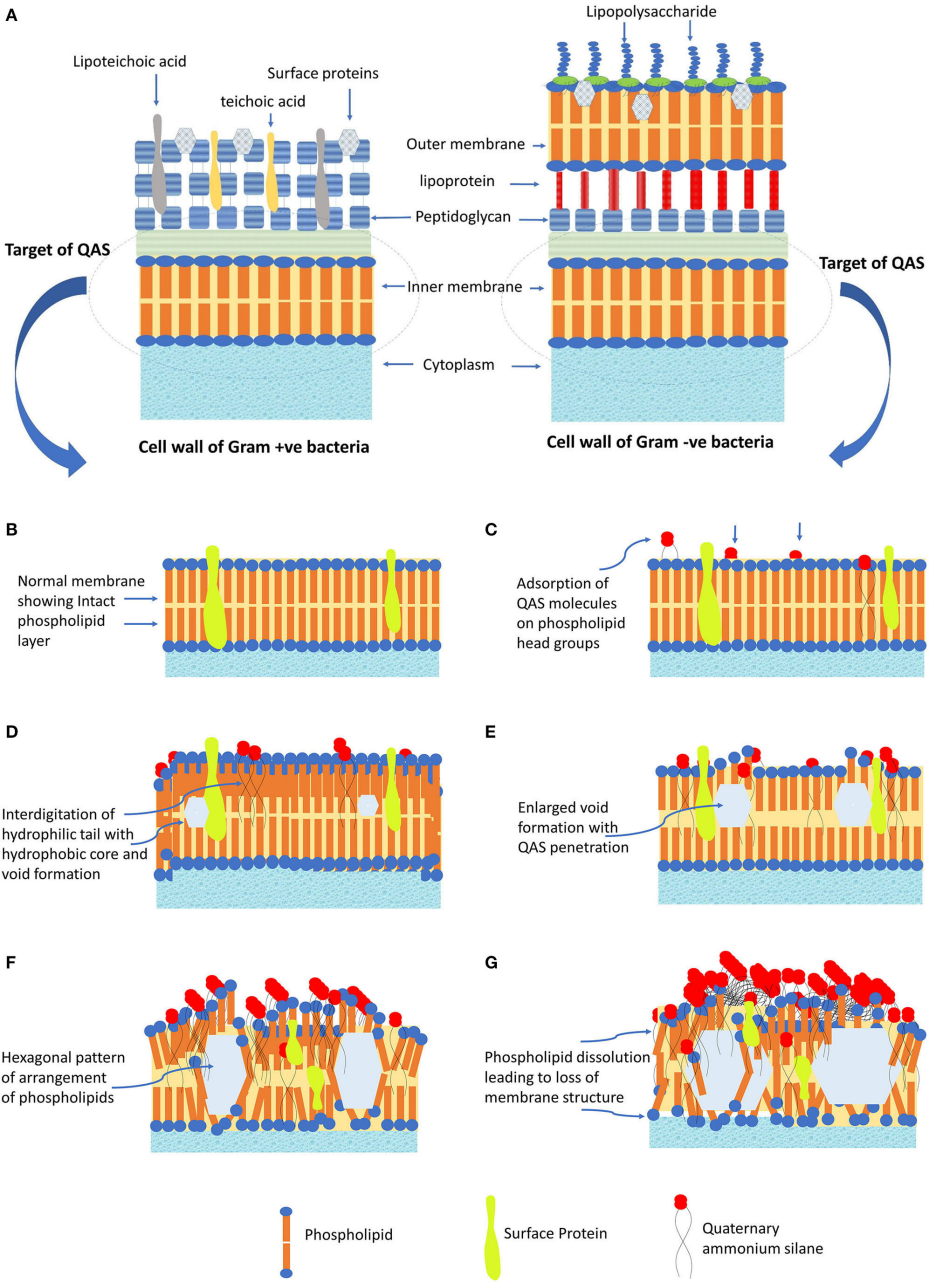
The antibacterial mechanism of QAS. **(A)** Cell membrane structure of Gram +ve and Gram -ve bacteria. **(B)** Normal structure of cell membrane, showing structure arrangement of phospholipids and surface proteins. **(C)** QAS molecules adsorbed onto the head group of membrane phospholipids. **(D)** Hydrophilic tail interdigitate into the hydrophobic core of membrane leading to void formation. **(E)** Increased penetration leads to enlarged void formation, causing further weakening of membrane. **(F)** Change of membrane structure to fluid crystalline state with a hexagonal pattern of arrangement. **(G)** Protein perturbation ensues and dissolution of phospholipid leading to loss of cell membrane structure and cell lysis.

The QASs are amphoteric surfactants, having one quaternary nitrogen connected with at least one major hydrophobic substituent. The n-alkyl chain length determines the lipophilic action of QAS (Gilbert and Al-taae, 1985). Generally, for quaternary ammonium compounds, maximum activity toward Gram +ve *bacteria* is seen with chain lengths of *n* = 12–14 and for gram –ve, *n* = 14–16. The mechanism of action QAS against bacterial cells is trepidation of lipid bilayer membranes that form the bacterial cytoplasmic membrane and the outer-membrane of Gram—ve bacteria (see Figures 2A,B). The strong antimicrobial function of QAS is due to –C_18_H_37_ lipophilic alkyl chain, which can penetrate the bacterial cell membrane causing its damage. QAS possesses positively charged nitrogen atoms, which are attracted toward the bacterial membrane that has cardiolipin and phosphatidylglycerol as negatively charged lipid moieties. Thus, QAS has a selective target of membrane lipids, leading to the lipophilic action of QAS (Alkhalifa et al., 2020). The K21 molecule adsorbs on the cell membrane with its cationic portion facing outwards and hydrophobic tails implanted into the lipid layer causing disorganization and leakage of low molecular components out of the bacterial cell wall (Ioannou et al., 2007). This leads to a total loss of structural organization of the cell, with loss of osmoregulation function (Rossi-Fedele et al., 2011; Daood et al., 2020b). K-21 exhibits “contact-killing action” by utilization of tetraethoxysilane as an anchoring component and having an organic functional group that are bound covalently together (Tiller et al., 2001).

To explain, at the molecular level, the mode of K21 action entails the involvement of a positively charged quaternary nitrogen with phospholipid acids in the membrane. The hydrophilic membrane core is penetrated by the hydrophobic tail (see Figures 2C,D). Exposure to QAS, consequently, causes an increase in the surface pressure and transformation of membrane from a liquid to a liquid crystal state creating voids (see Figures 2E,F). This leads to loss of physiological and osmoregulatory functions of the membrane. There is reduction of the hydrophobicity of the membrane core, and phospholipids tend to form hexagonal systems, generally occurring at MIC (see Figure 2G). QAS forms mixed micellar aggregates, which solubilize hydrophobic membrane constituents like phospholipids, lipid A. At higher concentrations, aggregates formed solubilize hydrophobic constituents of the membranes (Gilbert and Moore, 2005). This antibacterial action also depends on the pH, temperature, and the type of organisms used for testing (Kwaśniewska et al., 2020). Increasing the alkyl chain length leads to enhanced penetration into the bacterial cell membrane, disrupting similar to a needle bursting a balloon that leads to enhanced biocidal action (Tiller et al., 2001). Generally, the greatest biocidal activity indicates QASs with 10–12 carbon chains, while the increase and the decrease in the length of the alkyl chain diminish the antimicrobial activity (Kwaśniewska et al., 2020). The biocidal activity of QAS differs in action toward Gram-positive and Gram-negative bacteria. Research has shown that highest biocidal action of QAS for Gram-positive bacteria is with 12–14 carbon chains, while for Gram-negative is with 14–16 carbon chain. Also, counter-ions can have some effect on antimicrobial activity by altering the solubility of host polymers (Gilbert and Moore, 2005).

**Figure 3:**
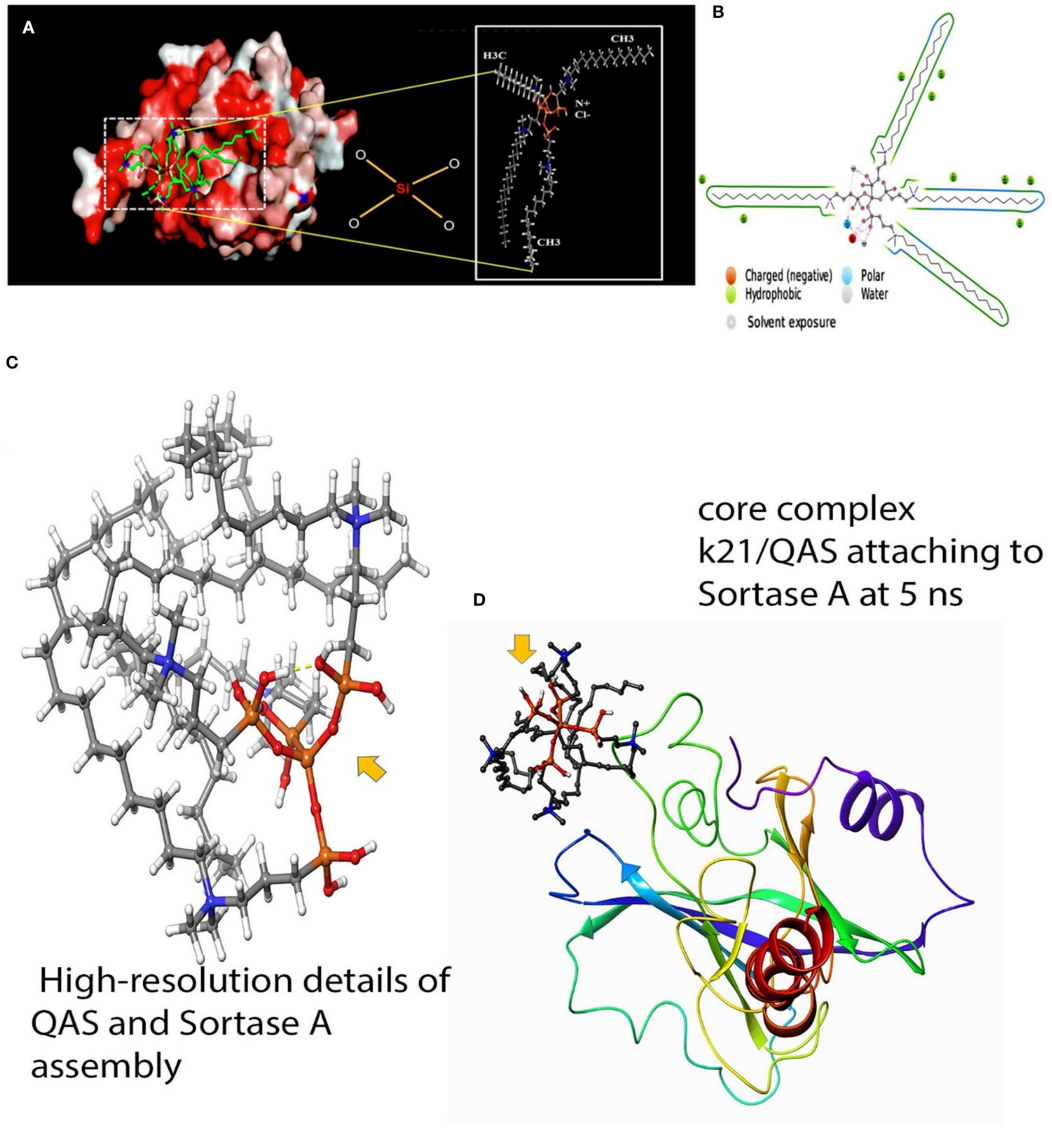
Molecular docking of QAS and srtA. **(A)** Results of molecular docking simulation of QAS 1% on crystal structure of SrtA, indicating a complex predicated interaction mode of the QAS catalytic center of SrtA. The structure was generated from molecular coordinates from the Protein Data Bank, PDB ID. The subset proposed chemical formula of the QAS molecule. The docking shown in the figure is typically performed on the basis of the known Sortase-A crystal structure and the SrtA-quaternary ammonium substrate complexes. The polar capabilities of QAS have enabled it to form charge-charge interactions that can insert with the binding pocket of SrT-A. **(B)** A schematic of detailed ligand atom interactions with the protein residues. Interactions that occur more than 5% of the simulation time in the selected trajectory (0.00 through 100.00 ns) are shown. Reprinted from: Daood et al. (2020b). **(C,D)** The figure indicates a schematic detailed ligand atom interaction with the protein residues of Sortase-A with the k21 molecule. K21 attaches and interacts within nanoseconds to prevent action of srtA.

Gram-positive bacteria like the *S. mutans* possess a distinct group of molecules having key adherent functions that help in initial steps of dental caries (Murata et al., 2010). In these bacteria, surface proteins are attached to the cell wall by Sortase A (SrtA) enzyme, coded with the SrtA gene, which recognizes the LPXTG motif (carboxylic leucine, proline, X, threonine, and glycine) of surface protein and catalyzes a sorting process to relate protein to the cell wall (Mazmanian et al., 2001; Paterson and Mitchell, 2004). SrtA plays a crucial role in the pathophysiology by regulating the capability of the bacteria to adhere to host tissue and expression of virulence (Huang et al., 2014). Thus, agents targeting SrtA enzyme can be beneficial as anti-adhesive agents in caries prevention. Molecular simulation has shown that QAS inhibits SrtA protein by causing conformational changes of the protein at the N- and C-terminal tail parts (see Figures 3A–D). Although molecular simulation depicts inhibition of SrtA, *in vitro* research is still lacking to validate use of QAS as an SrtA-inhibiting biomaterial. Thus, QAS can only be used as an SrtA inhibitor after evidence is obtained on its inhibitory mechanism on SrtA enzymes.

### Anti MMP Action

MMP play a vital role in formation of dentin matrix, modulation of caries progression, secondary dentin formation, tissue modeling, cell differentiation, and maintenance of homeostasis (Jain and Bahuguna, 2015). MMP-2 and MMP-9 structure has the N-terminal predomain, the pro-peptide domain, and a catalytic domain with a zinc-binding site (Jacobsen et al., 2010). An active site has two regions: a cavity has the zinc ion at the center and a specific pocket S1. Catalytic clefts possess six binding hydrophobic pockets that are S1, S2, and S3 (nonprime pockets on left side of zinc ion) and S1', S2', and S3' (primed pockets on the right side of zinc ion). The highest variable pocket is S1', while the lowest variable is S2' (Cui et al., 2017; Fischer et al., 2019). The S1' pocket determines the substrate specificity (Cui et al., 2017). As S1' pockets exhibit variability among all the MMPs, it is believed that this pocket can be a potential target for many MMP inhibitors (Berman et al., 2000; Fabre et al., 2014) like QAS agents. QAS displays inhibition of cathepsin-K and MMP-9 activity. This is due to binding of positive ammonium ions on negative glutamic acid residues, blocking the active site of MMP, preventing enzymatic degradation of dentin (Gou et al., 2018). Thus, QAS can be used as an alternative to 2% CHX as a cavity cleanser. QAS also exhibits improved dentin mineralization. These observations are related to inhibition of MMPs and cathepsins by QAS that enhances resistance to collagen degradation in dentin (Umer et al., 2017). Thus, QAS can be used as promising cavity disinfectant as it inhibits the protease enzyme and enhances the durability of resin-dentin bonds. Thus, combined anti-collagenolytic and antimicrobial action of QAS can be valuable in preventing degradation of resin dentin bonds and formation of secondary caries (Umer et al., 2017).

### Anti-inflammatory Action and Wound-Healing Capability of QAS

**Figure 4:**
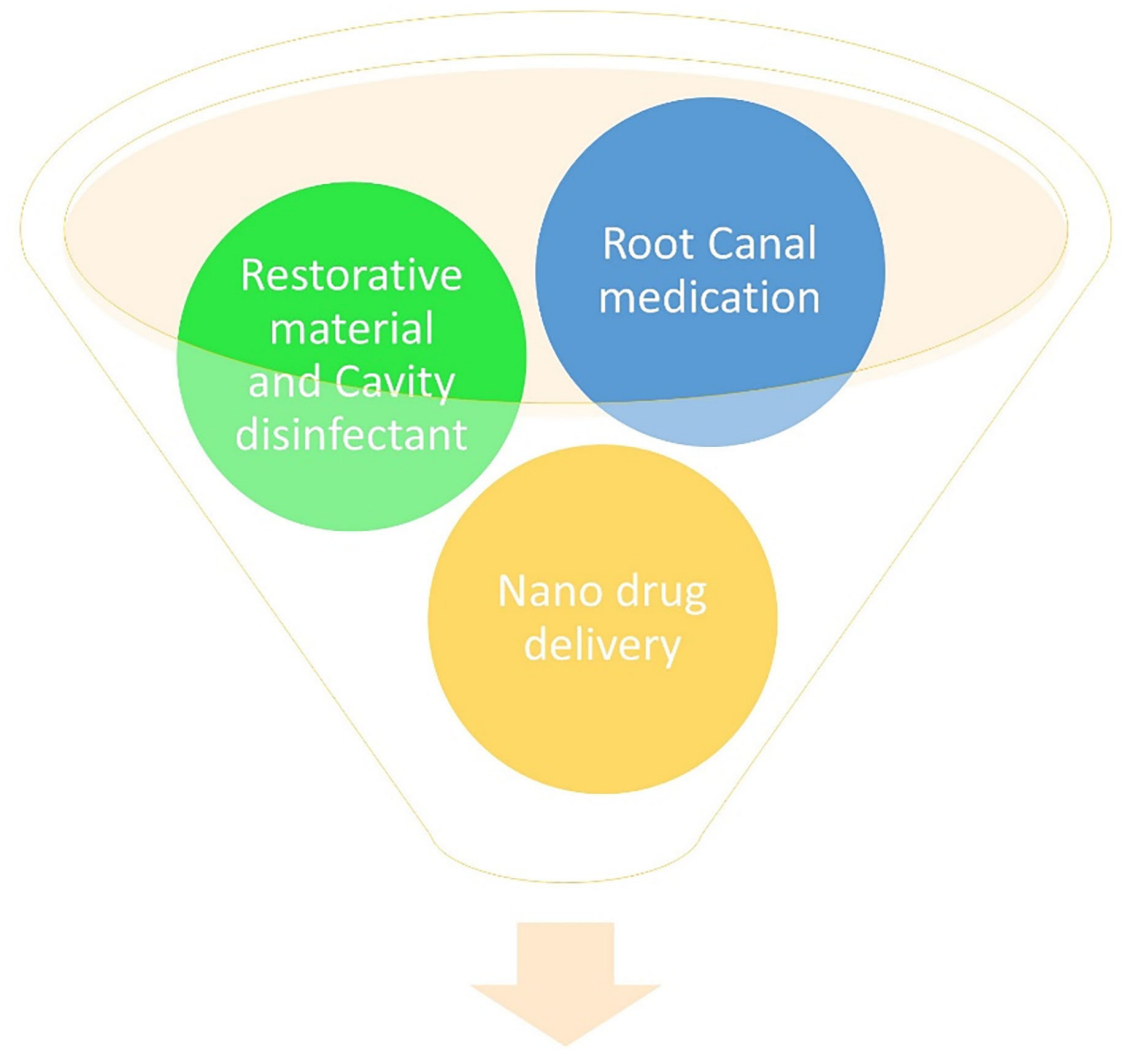
Application of QAS as a restorative material, cavity disinfectant, root canal medicament, and nano-drug delivery.

Macrophages are the subgroup of mononuclear cells that show response to implanted foreign material (Berman et al., 2000). Due to infection, these cells get activated and produce various cytokines and chemokines (de Almeida Neves et al., 2011). Their phenotype depends on cell markers, cytokine types, and functional characteristics of microenvironment (Walsh and Brostek, 2013). The polarized macrophages phenotypes are described as M1 and M2 profiles (Turkun et al., 2006). Macrophage provides an immense contribution during tissue healing and regeneration. The M1 phenotype produces proinflammatory cytokines [IL-1, IL-6, (TNF)-α], while the M2 phenotype depicts antiinflammtory molecules. M1 cells are involved in early and M2 cells in late inflammatory and tissue remodeling stages (Daood et al., 2017). During tissue repair, the macrophage phenotype switches from M1 to M2, which promotes anti-inflammatory effect while also stimulating the proliferation of keratinocytes, fibroblasts, and endothelial cells (Daood et al., 2022). Daood et al. depicted shown *in vivo* that Bone marrow stromal cells (BMSCs) obtained from Albino wistar rats were induced for M1 and M2 polarization along with Raman spectroscopy with a scratch assay. It was observed that QAS shifts the macrophage polarization profile from M1 to a, primarily, M2 anti-inflammatory phenotype, accelerating tissue repair and regeneration. Also, it was found that 0.5% k21 enhanced the wound-healing activity, with no visible wound area was found at the 24 h when compared to the vehicle control (Daood et al., 2022). In essence, QAS possesses unique properties that impact not only the oral microbiome but also the host immune-inflammatory response. QAs provides a novel biomaterial for tissue repair and regeneration of dental tissues; however, these features are translated into clinical dental applications, which are the focus of Section Applications of Quaternary Ammonium Silane as an Antimicrobial Biomaterial for Oral Microbiome (see Figure 4).

## Applications of Quaternary Ammonium Silane as an Antimicrobial Biomaterial for Oral Microbiome

### Restorative Material and Cavity Disinfectant

Although significant improvements have been attained in dentin bonding over the last few years, the resilience of tooth-colored restorations continues to be a clinical challenge that is yet to be addressed entirely. Secondary caries has been a major challenge in getting long-lasting resin–dentin bonds (de Almeida Neves et al., 2011). Although minimal intervention technique conserves tooth structure in management of deep carious lesions, yet viable bacteria retained within the dentin can lead to secondary caries and restoration failure over a period of time (Walsh and Brostek, 2013). Also, polymerization shrinkage from methacrylate-based resins causes secondary caries and restoration failure (Turkun et al., 2006). CHX has been used in restorative dentistry as an antimicrobial agent; however, the displacement of other cations of saliva and oral fluids by CHX compromises its antimicrobial action. Hence, there is a need for stable cavity disinfectant and a restorative agent that will reduce the chances of secondary caries and enhance the resin-dentin bond to make it more durable. In this section, current updates on the impact of QAS on restorative dentistry as a potent antimicrobial agent have been focused. Research has been done on application of QAS as a biomaterial to enhance the mechanical properties of restorations.

Zhang et al. prepared an experimental universal adhesive containing antimicrobial quaternary ammonium methacryloxy silane (QAMS) and evaluated its antibacterial action against *S. mutans* biofilm. Its effect on *S. mutans* biofilm was assessed by confocal laser scanning microscopy (CLSM), colony forming unit (CFU) counts, and the XTT assay. The experimental universal adhesive incorporating QAMS presented with significantly lower CFU counts of *S. mutans* than the control universal adhesive product (*p* < 0.001). This finding is supported by the Cell Proliferation Kit (XTT) cell metabolism assay, where the bacteria from the control group exhibited significantly higher metabolic activity (*p* < 0.05). After the burst release of leachable QAM in the 1st month, there is sustained contact-killing capability that inhibits the growth of *S. mutan*s along the adhesive-dentine interface. Moreover, the microtensile bond strength is not adversely affected by incorporation of QAMS into the adhesive system. The dual biocidal modes of the antibacterial adhesive system in the present study call for the incorporation of quaternary ammonium methacrylates into dentine adhesives. Quaternary ammonium methacrylates also have the advantage of inhibiting dentine-bound MMPs that are implicated in the degradation of the collagen network in the hybrid layer (Zhang et al., 2014).

Thus, QAMS presents a unique biomaterial synthesized by the sol-gel method and has flexible Si–O–Si bonds. Partially hydrolyzed resin having QAMS retains the antibacterial properties, preventing caries recurrence and maintaining of mechanical properties.

In another experiment, Gong et al. (2012) fabricated a visible light-cured co-monomers blend from bis-GMA and QAMS-3 and evaluated its antimicrobial and mechanical properties. The trifunctional QAMS was synthesized from SiQAC, TEOS, and 3-MPTS *via* a sol-gel process. The following characteristics for the co-monomer blends were determined: the degree of conversion, polymerization shrinkage, contact angle, cytotoxicity, dynamic mechanical properties, and its effect on the viability of biofilm cultivated from *S. mutans, Actinomycesnaeslundii (A. naeslundii)* and *Candida albicans (C. albicans)*. Based on the 3D-reconstructed biofilm images, it was observed that the biofilm cell mass directly adjacent to the resin surface was nonviable, with bacterial viability restored within the biofilm mass above this base layer. With increasing QAMS-3PH concentration, there is progressive reduction in volumetric shrinkage and cytotoxicity. The 70/30 mass percentage of bis-GMA/QAMS-3PH resin has similar biocompatibility as Teflon, which served as a control. QAMS-3PH containing resin exerts its antimicrobial action passively by contact killing. Microbes along the resin surface are readily killed upon contact due to the presence of surface-oriented long alkyl chains of the quaternary ammonium functionality. These antibacterial and antifungal activities are retained even after QAMS-3PH is co-polymerized with bis-GMA (Gong et al., 2012).

Thus, the above research shows that QAS can be used in restorative dentistry as an antibacterial agent, thereby increasing the longevity of restoration.

Application of a cavity disinfectant prior to restorative procedures enables anti-proteolytic and antimicrobial effects, thereby preventing formation of secondary caries and hybrid layer degradation (Gou et al., 2018). Gou et al. evaluated the growth of bacteria impregnated within dentine blocks and the gelatinolytic activity of the resin-dentine interface, following treatment with a 2% QAS cavity cleanser. CLSM and CFU measurements were used to assess its antimicrobial activity. The hybrid layer was subjected to *in situ* zymography to analyze its gelatinolytic enzyme activity. The researchers also looked into the inhibitory effect of the cavity cleansers against rhMMP-9 and cathepsin K. Dentine treated with 2% QAS, and 2% CHX had significantly better antimicrobial effect than the control without a cavity cleanser (*p* < 0.05). The difference between 2% QAS and 2% CHX, however, was not statistically significant. A similar pattern was observed for intratubular gelatinolytic activity, which was significantly lesser after pre-treatment with either QAS or CHX. Disinfection with 2% QAS also rendered the interface less water permeable with a relative permeability of 53.7 ± 4.8%. Through its four positively charged quaternary ammonium arms and four long, lipophilic C18 alkyl chains, QAS is able to attach to the negatively charged bacterial cell wall. This electrostatic interaction allows the long alkyl chains to penetrate the cell wall and membrane, causing release of cytoplasmic contents and, finally, cell death (Gou et al., 2018). In another research, Daood et al. (2019) assessed the effect of QAS on cariogenic bacteria to be used as a cavity disinfectant. Different concentrations of QAS (2, 5, and 10%) were compared with 2% CHX against *S. mutans, L. acidophilus*, and *A. naeslundii* grown on dentin disks. These were grown as single, dual, and multispecies biofilms. Similar to previous studies, the effect of disinfectants was assessed by CFU, 3-(4,5-dimethylthiazol-2-yl)-2,5-diphenyl tetrazolium bromide (MTT), CLSM, and Raman spectroscopy. CLSM showed the highest dead cell count depicted by red color staining was for 10% QAS conc. Thus, QAS's antimicrobial effect was concentration dependent. Although the log of CFU count was decreased for disinfectants compared to control, there was no significant difference in CFU for single and dual species biofilms after treatment with different concentrations of disinfectants. Thus, increase in concentration of the disinfectant had no effect on log CFU of biofilms. The MTT assay showed that, with an increase in concentration and exposure time, there was a decrease in metabolic activities of *S. mutans* and *L. acidophilus*. With increase in QAS concentration, C-H alkyl, and Amide I and III groups depicted a shift with high frequencies. The shifts observed may be due to sensitivity of the polypeptide chain. A CHX-treated specimen depicted lesser intensity in the same regions. QAS interaction with demineralized dentin led to chemical changes in tooth surfaces and eliminated the cariogenic bacteria. Thus, QAS can be used as an antimicrobial cavity disinfectant before application of adhesives (Daood et al., 2020a).

Similarly, Daood et al. (2019) analyzed the effect of QAS cavity disinfectant on transdentinal toxicity and phenotypic response of macrophage. Of all the concentrations (2, 5, and 10% QAS, 2% CHX, deionized water as the control group) tested, 2% QAS did not exhibit cytotoxicity on NIH 3T3 mouse fibroblast cells. Approximately, 2% QAS showed no detrimental effect on total protein and mineralized nodule formation, although alkaline phosphatase was slightly reduced in quantity compared to control. Alkaline phosphatase has a vital role in mineralization of dentin matrix (Golub and Boesze-Battaglia, 2007). Approximately, 2% QAS did not affect nodule production, indicating that it can help in formation of reparative dentin function that is protective in nature. Increase in concentration of QAS (5 and 10%), 2% CHX-depicted reduced protein, a mineralized module, and alkaline phosphatase production show concentration-dependent deleterious effect. This toxicity was found to be concentration dependent with 2% QAS, showing the best results. Use of 2% QAS depicted mainly M2 phenotypic expression suggestive of anti-inflammatory effect, enabling tissue repair. Thus, *in vitro* results proved that 2% QAS can be used as a cavity disinfectant due to its non-cytotoxic effects on mouse fibroblasts and anti-inflammatory expression, showing a reparative effect (Daood and Yiu, 2019).

**Figure 5:**
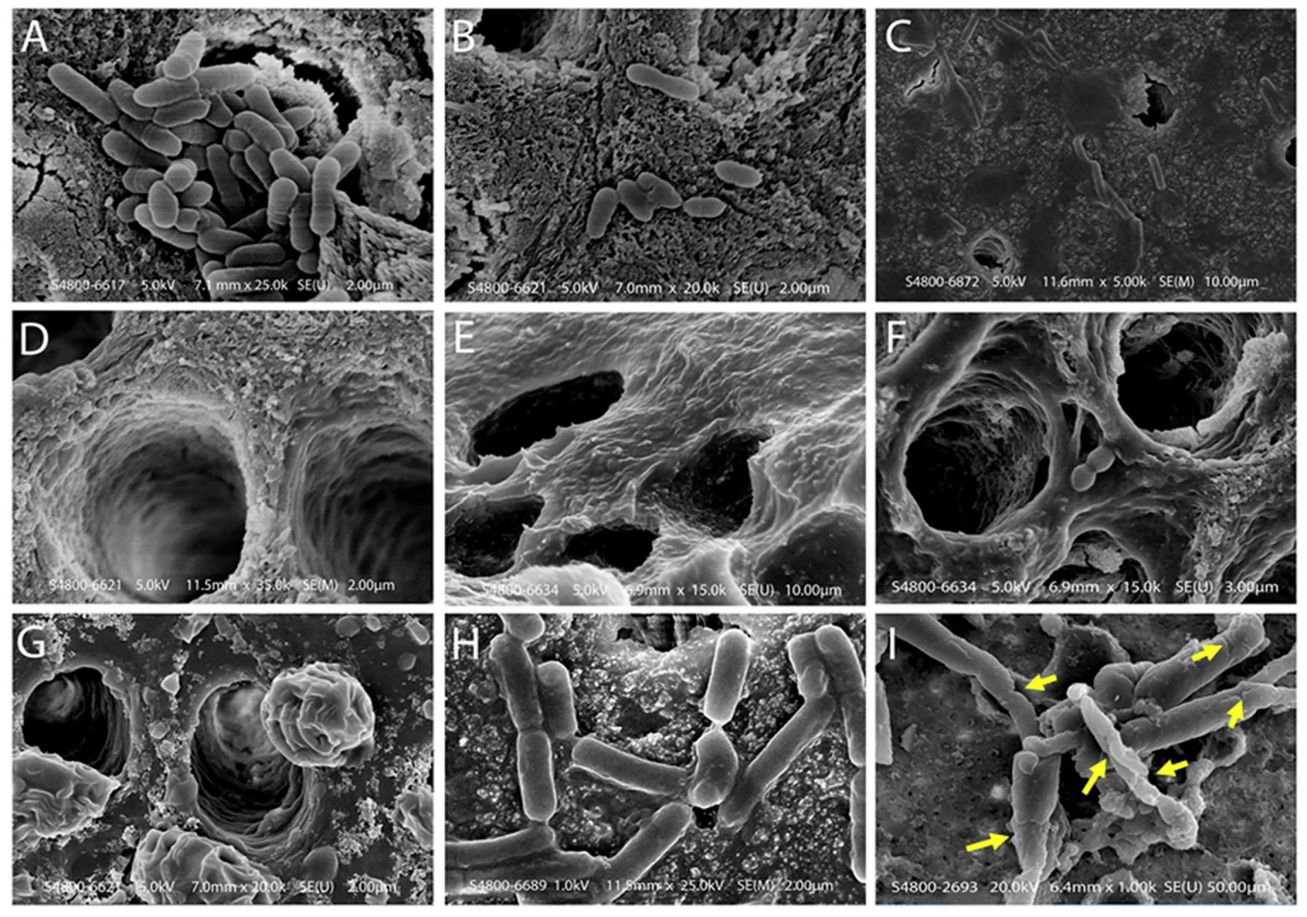
Scanning electron microscope images of dentin samples treated with biofilm and test solutions. **(A)** A scanning electron microscope of a control specimen, showing dentinal tubules covered with the dual species biofilm. Bacteria and debris are present on the dentine surface without using standard experimental disinfection. Bacteria blocked the opening of the dentinal tubules; groups displayed singular or multiple deposits on the sample with bacterial cells clumping and chaining to form complex biofilms. **(B)** SEM showing incomplete removal of bacteria on the dentine surface after using 2% CHX protocol. These dentinal tubules are located in the middle third of the dentine specimen. There were small colony chain formations seen among 2% CHX specimens **(B)** due to slight restructuring as compared to maximum detachment seen in QAS groups. **(C,D)** Bacterial penetration is limited across the lengths of dentinal tubules and the dentinal surface demonstrated in 1% and 2% QAS specimens. **(D)** The tubule wall of demineralized dentine treated with 1% QAS shows exposed fibrillar collagen network. **(E,F)** Representative SEM images of etched dentine following application of 1% and 2% QAS, respectively, showing the QAS molecules did not completely infiltrate into the demineralized collagen matrix, forming a crust on the surface. A phase separation is seen due to the presence of water **(G)**. **(H)** Bacterial biofilms were generally intact within control specimens; **(I)** the bacterial Lactobacillus within the biofilm showed rough and wrinkled surfaces observed on the membrane after treatment with 1% QAS. There were large, damaged areas, including the formation of holes inducing significant damage to the membrane of bacterial cells after use of 1% QAS **(I)**. Reprinted from Daood et al. (2020b).

Daood et al. (2020a,b,c) investigated the antimicrobial action of various concentrations of QAS on *S. mutans* and *Lactobacillus acidophilus* single and dual species biofilms. SrtA protein had conformational changes because of the QAS molecule as shown by simulation. Scanning Electron Microscopy (SEM) showed limited bacterial penetration into dentinal tubules (see Figure 5). Approximately, 1% QAS-treated dentin depicted collagen fibrillar architecture. Raman spectroscopy showed that peaks looked dull, and low carbohydrate intensities (480–490 cm^−1^) for 1 and 2% QAS showing biofilms were affected at lower concentration of 1% QAS. DAPI staining used to assess the DNA damage showed that less-stained DNA remained within the cell, showing leaking of intracellular constituents along with DNA from damaged membrane of QAS-applied specimens. This is suggestive of bursting of the cell wall because of osmotic pressure created due to the action quaternary ammonium compounds (Melo et al., 2011). In the QAS molecular structure, altering the alkyl chain length determines the threshold of its antimicrobial action. There was an increase in fatty acid and SDH levels of biofilms for 1 and 2% QAS specimens suggestive of membrane damage action of QAS, which is concentration dependent. Thus, 1% QAS can be used as an effective cavity disinfectant for microbial elimination (Daood et al., 2020b).

Thus, the research data demonstrate that QAS can be used as a prospective antimicrobial cavity disinfectant in restorative dentistry. It eliminates caries-causing bacteria from tooth cavity and possess potential to overcome the drawbacks in relation to other available disinfectants.

#### Impact of QAS on Bond Strength in Adhesive Systems

Daood et al. observed the effect of QAS as a cavity disinfectant and on durability of dentin bond strength. There was no significant difference in the nanoleakage score in the 2% QAS group between the baseline and 1 year of both the adhesives. Nanoleakage was significantly more in 5% QAS, 2% CHX, and the control group, Thus, dentin pre-treatment with 2% QAS prevents degradation of a bonded interface. For both the adhesive systems Prime and Bond1 NTTM (PB, Dentsply DeTrey, 78467 Konstanz, Germany) and AdperTM Single Bond 2 (SB, 3M ESPE, St. Paul, MN, USA), there was no significant difference in tensile bond strength in groups pre-treated with 2% QAS, 2% CHX, and control. AS QAS concentrations increased (5 and 10%), there was reduction in bond strength. This could be due to the condensed QAS layer that may act as a stress raiser, causing cracks and microfractures, leading to composite layer detachment. Also, presence of higher concentration of QAS within demineralized dentin can affect polymerization of adhesive systems affecting their mechanical properties. Inadequate polymerization of the adhesive resin can lead to the weakened hybrid layer, reducing the bond strength and durability over time (Daood et al., 2017).

Gou et al. investigated the influence of the 2% QAS cavity cleanser on bond strength and showed that QAS did not impact bond strength of adhesive. This could be due to formation of network between resin and polysiloxane at the time of polymerization of adhesive resins. QAS also enhances hydrophobicity of the dentin surface, causing increased wetting of the demineralized surface and penetration of adhesives. During silanol group hydrolysis, as water is consumed, reduction in water helps for infiltration of resin and enhances polymerization of adhesive. All these reasons contributed to good bond strength (Gou et al., 2018).

Daood et al. investigated effect of novel 2% quaternary ammonium silane (QAS) as an on-dentin substrate. Approximately, 6% NaOCl + the 2% CHX group showed completely obliterated dentin tubules, with thick deposits suggestive of formation of para-chloroaniline (PCA) deposits due to interaction of NaOCl and CHX. SEM analysis of the resin-sealer interface showed that resin tags penetrated deep into the tubules as smooth rods in 6% NaOCl + the 2% QAS group compared to other groups. This is due to the organosiloxane group of QAS has high affinity for resin adhesives. It also causes higher wettability by increasing the hydrophobicity of the dentin surface, leading to increased resin penetration (6). Thus, there is a direct correlation between the type of irrigant solution and penetration of resin tags. Thus, novel 2% QAS when used in combination with 2% CHX can be used as an effective antimicrobial irrigant for root canal infections (Daood et al., 2019). Thus, QAS can be used a potential cavity applicant for integration into the resin–dentine-bonding protocol.

### Root Canal Medication

Microorganisms invade the root dentin by acidic attack *via* cracks in dentin, leading to pulpal infection. These bacteria can repopulate the root canals and can cause endodontic failure (Siqueira, 2001). As per the current guidelines, abundant irrigation is required to disinfect the root canals removing the bacteria (Dahlkemper et al., 2016). CHX has been used in root canal disinfection; however, it is water soluble with reversible bonding among protonated amine groups that lead to its leaching out from bonded interfaces (Gendron et al., 1999; Breschi et al., 2010). It also loses its antimicrobial potency due to chelation by calcium from saliva. NaOCl when used with CHX forms para-chloroaniline that prevents the sealing in obturation and blockage of lateral canals (Basrani et al., 2010). Hence, this review discusses studies done on formulation of QAS as a novel root canal medication. It has been tested for bacterial elimination from root canal system.

Daood et al. (2019) investigated effect of novel 2% QAS as an endodontic irrigant for its antimicrobial efficacy and effect on dentin substrate. Groups with combination were used for the study: 6% NaOCl + 2% QAS, 6% NaOCl + 2% CHX and saline. Raman spectroscopy, CLSM, and SEM were used for analysis. CLSM results revealed the *E. faecalis* biofilm treated with QAS exhibited red fluorescence, indicating that most of the *E. faecalis* biofilms were dead. This action is due to the positively charged quaternary amine (N+) that interacts with negatively charged bacterial membrane, causing contact killing. Due to the presence of silanol groups, QAS bonds covalently by O-Si-O linkages to employ nonmigrating microbicidal action. Raman spectroscopy depicted a decrease in intensity in 484 cm^−1^ regions of biofilms in 6% NaOCl + the 2% QAS group (compared to 6% NaOCl + 2% CHX – 1,350–1,420 cm^−1^ region), suggesting decrease in carbohydrate content with lower number of bacterial colonies. SEM observed thin crust on the dentin surface and absence of *E. faecalis* colonies in 6% NaOCl + the 2% QAS group, suggesting maximum lysis and eradication of the *E. faecalis* biofilm. Approximately, 6% NaOCl + the 2% CHX group showed completely obliterated dentin tubules with thick deposits suggestive of formation of para-chloroaniline (PCA) deposits due to interaction of NaOCl and CHX. Thus, novel 2% QAS when used in combination can be an effective irrigation protocol for root canal infections (Daood et al., 2019).

Daood et al. (2020a,b,c) experimented on QAS in order to determine its antibacterial action against *E. faecalis*, its cytotoxicity levels, and possible interferences with the mechanical properties of the dentine substrate. The irrigation protocols are divided into 6% NaOCl, 6% NaOCl + 2% CHX, 3.5% QAS, 2% QAS, and sterile saline as the control. Their antibacterial strength was assessed with Raman spectroscopy, CLSM, and the CFU log of the *E. faecalis* biofilm. QAS irrigation was accompanied by a significant increase in the dead *E. faecalis* biofilm, with the 3.5% concentration capable of killing the majority of cells (86.7 ± 4.3). On the other hand, QIS specimens showed increased cellular proliferation compared to other irrigants. The original cellular morphology was more or less preserved, with increased spread of fibroblastic cells on root dentine. Soon after application, QIS exerts a strong and rapid antibacterial action. After this burst of activity has subsided, the QIS crust formation remains active and available for contact killing that lasts up to 7 days, thus preventing the regrowth of bacteria. Despite the aforementioned crust deposition onto the root dentine surface, resin infiltration into the exposed tubules was unperturbed. It is theorized that resinous affinity of the organosiloxane group of QAS contributed to better penetration of the resin sealer (Daood et al., 2020c).

**Figure 6:**
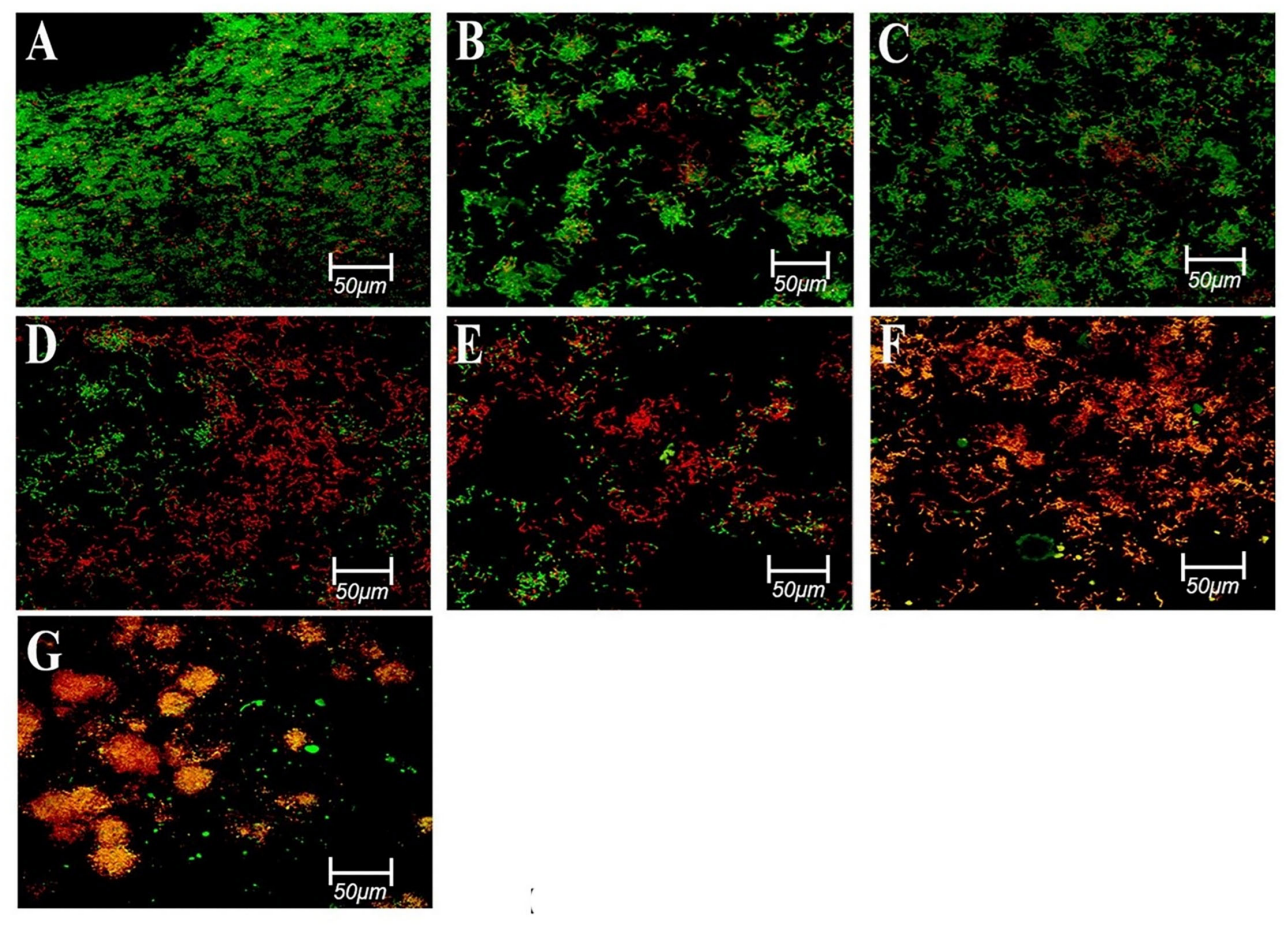
Representative confocal images of *E. faecalis* biofilms of different groups stained using a live/dead bac light bacterial viability kit; **(A,B)** control after 3 days; **(C)** control after 7 days; **(D)** 2% CHX after 7 days; **(E)** 41% Ca(OH)2 after 7 days; **(F)** 2%k21 after 3 days; and **(G)** 2%k21 after 7 days. Excitation was performed at λ = 514 nm. Green indicates a high level of bacterial viability in control specimens. Most of the red fluorescence in 2%k21 specimens indicating dead cell population. Scale bar = 50 μm. Reprinted from Kok et al. (2021).

In another study, Kok et al. (2021) compared the susceptibility of *E. faecalis* and *C. albicans* biofilms to the antimicrobial action of different intracanal medicaments, namely, quaternary ammonium silane (QAS)/k21, CHX, and calcium hydroxide. The k21 intracanal medicament was prepared *via* the sol-gel method. Dentine blocks contaminated with *E. faecalis* and *C. albicans* were subjected to CFU counting, CLSM, and Raman analysis for microbial assessment. All three intracanal medicaments completely suppressed the growth of *E. faecalis* and *C. albicans* at 200- and 400-μm depth of dentinal tubules. There is a significant reduction in the *E. faecalis* biofilm (*p* < 0.05) when 2% k21 is used in lieu of CHX to treat the root dentine (see Figure 6). For *C. albicans*, the best antimicrobial action was found in calcium hydroxide, followed by 2% k21 and, finally, CHX. TEM images showed that root dentine treated with 2% k21 had increased number of collagen fibrils arranged in a relatively uniform manner. It is proposed that 2% k21 has a prolonged antimicrobial effect without an apparent decline over time. This is due to the release of SiQAC molecules forming a protective film that is capable of circumventing and hindering the growth of biofilms (Kok et al., 2021). Thus, the above research shows that QAS can be a more efficient substitute to NaOCl and CHX as a root canal irrigant, considering its sustainable antibacterial action.

A new class of insoluble macromolecular disinfectants has been developed, which can directly inactivate target microorganisms without releasing any antibacterial agents into the system (Kenawy et al., 2007). The mechanism is based on the positive charge of the macromolecules interacting with the negatively charged bacterial membrane, causing it to be disrupted. Various nanoparticles, such as Chitosan, Ag, and quaternary ammonium polyethyleneimine (QA-PEI), have been added to irrigants, intracanal medications, and sealers as a result of this approach (Kishen et al., 2008; Shrestha and Kishen, 2014). A new epoxy resin sealer (BJM ROOT CANAL SEALER^®^, BJM Laboratories Ltd., Or-Yehuda, Israel) contains BioSafe^®^ (HM4100, BIOSAFE Inc., Pittsburg, PA, USA). This substance satisfies the ISO standard. The Biosafe^®^-incorporated epoxy resin sealer was tested for its *in vitro* antibacterial effect against the existing biofilm. The higher Biosafe^®^ concentrations (1.6 and 3.3 percent w/v, respectively) resulted in significant reductions in biofilm viability (20 and 36%, respectively). As a result, Biosafe^®^ may enhance the antibacterial effect of the root canal sealer by influencing the residual biofilm (Becker et al., 2019).

### Nano-Drug Delivery

Nano-drug delivery systems are a type of nanomaterials that have the capability to potentiate the water solubility of drugs, enhance the drug uptake, increase the cycle time, and decrease enzyme degradation, thus improving the effectiveness and safety of drugs (Gupta et al., 2019). Researchers have used various approaches, such as proliposomes, polymeric-micelles, PLGA-PEG, and emulsions, to decrease the regulating aspect of bioavailability and delivery (Xu et al., 2016). Poly (lactic-co-glycolic acid) (PLGA) is a comprehensively studied polymer certified by the US Food and Drug Administration (FDA) for medical purposes (Pandey and Jain, 2015). PLGA nanoparticles have demonstrated to be a possible drug delivery system (Dalpiaz et al., 2016) and are good biodegradable copolymers decomposing into non-toxic by-products that are removed from the body. This section discusses about the research done on nanoparticles as carriers for delivery of k21 biomaterial for antibacterial effect and improvement of bond strength.

Bai et al. (2019) developed QAS-grafted hollow mesoporous silica (QHMS) to formulate metronidazole-sustained delivery system (MDZ@QHMS), with a bimodal killing mode. MDZ@QHMS showed sustained drug release and bactericidal action against *Staphylococcus aureus* (*S. aureus*), *Escherichia coli* (*E. coli*), and *P. gingivalis* at concentration of 100 μg/ml and above. This newly developed delivery system was not cytotoxic to fibroblasts below 100 μg/ml. MDZ@QHMS displayed antibiotic elution and bimodal contact killing antimicrobial activities. Thus, a high-loading volume of HMS nanoparticles, presence of silanol groups on silica for availability of functional groups, and biodegradable silica provide MDZ@QHMS as a novel sustained delivery system with a bimodal killing action (Bai et al., 2019). Such types of nano-delivery systems may be effective in local drug delivery in periodontal pockets to eliminate the oral biofilms and prevent the recurrence of the disease.

Fan et al. (2020) developed PLGA submicron particles loaded with k21, calcium, and phosphorous (P-CaPK) and assessed their antimicrobial and mineralization potential *in vivo* and *in vitro*. P-CaPK showed dose-dependent *in vitro* antimicrobial action against *E. faecalis*. These submicron particles on ultrasonic activation were able to infiltrate dentin tubules and exhibit antibacterial action. This could be due to the smaller particle size along with positive zeta potential. P-CaPk showed better *in vitro* mineralization potential compared to particles loaded only with K21. These observations are related to the inhibition of MMPs and cathepsins by k21 that enhances resistance to collagen degradation in dentin (Umer et al., 2017). Ca, P, and Si are essential for *in vitro* and *in vivo* osteogenesis and mineralization (Hinata et al., 2017; Li et al., 2017). Si in k21 acts as a nucleation base for Ca and P for mineralization. Thus, formation of apatite crystals on the canal wall blocks the dentin tubules, preventing bacterial infiltration and restoration of periapical defects due to resorption. P-CaPk showed prevention of *E. faecalis* infection in root canals of beagle dogs. Thus, research by Fan et al. showed that K21 has good biocompatibility, antimicrobial action, and *in vitro* mineralization and is an effective disinfectant in root canal treatment (Fan et al., 2020).

Daood et al. (2021a,b) developed novel K21 PLGA nanoparticles with VE-TPGS and riboflavin-5-phosphate solution (Nano-PLGA⋮RF/VE-TPGS0.50 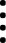 k21) to assess it as a delivery tool at the demineralized dentin surface. It was assessed for its chemical interaction, structural integrity, antibacterial resistance, and mechanical characteristics. Smooth textured spherical nanoparticles with particle size < 200 nm and with high k21 concentration were prepared. This novel nanoparticle exhibited positive zeta potential and increased colloidal size. Dentin specimens treated with Nano-PLGA⋮RF/VE-TPGS0.50 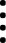 k21 exhibited disintegration of biofilms, nonviable cells showing antibacterial action. The action was seen to be intratubular with nanoparticles flowed through dentin tubules with adhesive impregnation to eradicate bacteria. Thus, these nano-PLGA⋮RF/VE-TPGS0.50 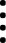 k21 infiltrate deep inside the dentin tubules. They also reduced exopolysaccharides production and caused detachment of biofilms from surfaces. *Ex vivo* results showed that it is biocompatible to human mesenchymal stem cells and could be due to increased nanoparticle concentration with drug encapsulation. The resin-dentin adhesive interface depicted development of a well-formed hybrid layer with adequately formed resin tags after use of etch and rinse-bonding protocol to demineralized the dentin surface treated with Nano-PLGA⋮RF/VE-TPGS0.50 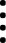 3%k21. Lower intensity of Raman bands for Nano-PLGA⋮RF/VE-TPGS0.50 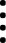 1%k21 and 3%K21 compared to control indicates damage to nucleic acid of bacterial cells. There was decrease in hydroxyproline liberation in groups treated with Nano-PLGA⋮RF/VE-TPGS0.50 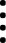 3%k21, depicting higher resistance to collagen degradation. Thus, because of K21 exhibits better dentin penetration, enhanced resin-dentin bond strength, excellent biocompatibility and preservation of collagen structure, PLGA loaded with k21 can be considered an innovative mode of drug delivery in adhesive dentistry (Daood et al., 2021a). However, for k21 loading, about 100 mg of PLGA⋮RF/VE-TPGS0.50 was directly dissolved in dichloromethane at 50°C. Upon obtaining a clear solution, different concentrations of k21 were added to the solution (~1 wt%, ~2 wt%, and ~3 wt%). Because of the presence of long hydrocarbon chains, k21 increases hydrophobicity of demineralized dentine by changing its surface energy. Moreover, the reduction in cell viability could also be ascribed to increasing nanoparticle concentrations and drug encapsulation as observed with PLGA⋮RF/VE-TPGS0.50 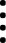 3%k21. This may be a far-ranging factor because demineralization depth limits deep penetration of nanoparticles and prevents it from contacting a pulp (Daood et al., 2021a,b). However, the overall crucial part of this study was to indirectly investigate the effect of released k21 from the nanoparticles, a concept that needs further research and evaluation.

Nikolaidis et al. synthesized novel nanocomposite system having polymerizable QAS-modified silica nanoparticles. Addition of silica nanoparticles improved the flexural strength and compressive strength. Polymerization shrinkage was reduced by addition of nanoparticles (Nikolaidis et al., 2021).

The antecedent summary strongly supports the use of QAS in various aspects of dentistry to optimize the outcomes of direct restoration and root canal treatment. Besides conventional dental treatment, QAS has been used as active constituents in contemporary therapeutic tools, such as nano-drug delivery system, which bodes well for the future of this antibacterial agent.

## Discussion

Resin-based restorative materials based on bis-GMA formulations are susceptible to failure, most commonly due to secondary decay and fracture of restoration (Sarrett, 2005). These could potentially be counteracted by the passive, contact-killing antimicrobial activity exhibited by the QAMS-3-blended resin, which also presented with increase in the degree of monomer conversion, reduction in polymerization shrinkage, enhanced fungicidal activities, and decrease in cytotoxicity (Gong et al., 2012). The reduction in volumetric shrinkage without adversely impacting on the monomer conversion of QAMS-3 containing resin matrices could be caused by a higher rate of free radical diffusion because the flexible siloxane backbone imparts increased polymer chain mobility (Owen, 2002). Furthermore, the antifungal activities of QAMS-3 against *C. albicans* can be leveraged for the design of antibacterial/antifungal denture bases by copolymerizing polymethyl methacrylate with QAMS-3. This could prevent malodor and mucosal inflammation that are so often found in denture patients or orthodontic patients wearing retainers (Dhir et al., 2007).

The formulation of antibacterial QAMS by Zhang et al. deserves to be mentioned as they substituted the tetrafunctional TEOS anchoring unit with dimethyldiethoxysilane (DMDES), given that the former was associated with limited solubility in solvents, such as methanol, ethanol, and acetone. It should be stressed that, following inclusion of DMDES-derived QMAS, the bond strength of the adhesive to dentine was not weakened. Another theoretical advantage is better stress dissipation due to the bending flexibility of the Si-O-Si bond angle.

Addition of QAS on the pre-treated acid-etched surface enhances surface hydrophobicity by altering the surface energy of the dentine surface. Improved surface energy would cause increased wettability of the demineralized surface and greater penetration of the adhesive resin monomers (Lung and Matinlinna, 2010). Organofunctional silanes that are also water scavengers interact with water molecules, hydrolyzing alkoxy groups connected to the silanes and convert them to alcohol molecules. The inclusion of hydrophobic QAS into the bonded surfaces results in the development of a new hydrophobic and durable hybrid layer. Decrease of residual water within the demineralized dentine will expedite infiltration of the adhesive resins, causing improved encapsulation of the collagen fibrils, enhanced bond durability (Hosaka et al., 2009). Hence, 2% QAS can be used as a cavity disinfectant with negligible effects on the bond strength of the simplified etch and rinse adhesives, and efficiently protects the strength of the hybrid layer and the bonded interface with time.

Both 2% QAS and 2% CHX cavity cleansers exerted similar inhibitory effect against the activity of cariogenic bacterial biofilm and intratubular gelatinolytic activity. In addition, unreacted QAS monomers may continue to form siloxane bridges within the silicate network, extending its antibacterial effect even after adhesive application (Chen et al., 2017). These benefits are of great relevance because secondary caries and degradation of the hybrid layer are two major challenges in achieving a durable resin-dentine bond (de Almeida Neves et al., 2011; Tjäderhane et al., 2013).

It was mentioned earlier that 2% QAS dilution significantly inhibited SrtA protein by inducing conformational changes of the protein, particularly at the N- and C-terminal tail parts. Since SrtA is involved in the modulation of surface properties of bacteria that mediate tooth surface adherence, its inhibition will reduce the cariogenicity of *S. mutans* (Lee and Boran, 2003). Reduced DAPI staining following treatment with 1% QAS, DAPI implied that there was lesser double-stranded DNA within the remaining cells (Daood et al., 2020a,b,c). This was likely the result of the leakage of DNA and other intracellular contents from damaged cell membranes in specimens treated with QAS.

Despite an apparent dose-response relationship in the antibacterial efficacy of QAS, time was an important factor to be considered, as the metabolic activity of *S. mutans* and *L. acidophilus* biofilms decreased with increasing exposure time to cavity disinfectants. Despite the effort to simulate the *in vivo* complex microbial ecosystem by preparing multi-species biofilms of two–three common cariogenic species, there are still considerable differences compared to the oral cavity's complex polymicrobial plaque biofilm in terms of survival and pathogenicity. Hence, there is still work to be done before clinical recommendations can be made for the use of a QAS cavity disinfectant during caries management.

The role of QAS in managing root canal is of great interest to the dental community. Infected root canals are colonized by a complex microbiota, chief of which is *E. faecalis* (Zhang et al., 2015), which can survive in the harsh conditions of high alkalinity and proven to be resistant against many antimicrobial agents (Saleh et al., 2004). As such, QAS shows promise as an alternative antimicrobial agent in endodontic practice, as QIS led to significant reduction of live bacteria in the *E. faecalis* biofilm, with the concentration of 3.5% QAS removing most of the bacterial cells. The crust formation following QAS irrigation was biologically active, involved in prolonged contact killing that lasted 7 days, which prevented bacterial repopulation. In contrast to NaOCl irrigation that demonstrated degradation of the collagen matrix, the collagen fibers in QAS specimens remained intact with evident cross banding. This is because QAS is known to promote stabilization, resulting from the formation of molecular siloxane bridges. Moreover, the silicon (Si-C) within the silane network acts as a nucleation site for apatite crystallites that may aid in dentinal tubule occlusion and, in turn, prevent bacterial invasion (Dong et al., 2011). When used in combination with other irrigants, 6% NaOCl + 2% QAS exhibited good antibacterial activity against the *E. faecalis* biofilm, as indicated by mostly red fluorescence in confocal laser microscopic images. Thin crust deposition on the dentin surface and absence of *E. faecalis* colonies in 6% NaOCl + the 2% QAS group suggested maximum lysis and eradication of the *E. faecalis* biofilm. On the other hand, 6% NaOCl + the 2% CHX group showed thick deposits suggestive of formation of para-chloroaniline (PCA) due to interaction between NaOCl and CHX. SEM analysis of the resin-sealer interface showed that resin tags penetrated deep into the tubules as smooth rods in 6% NaOCl + the 2% QAS group compared to other groups. In contrast to the para-chloroaniline crust deposition when NaOCl and CHX irrigation are used sequentially, the QIS crust formed did not inhibit resin penetration into exposed dentinal tubules. This is because the organosiloxane group of QAS has high affinity for resin adhesives (Lung and Matinlinna, 2010). Thus, there is a direct correlation between the type of irrigant solution and penetration of resin tags. Moreover, NaOCl and 2% QAS irrigation protocol had negligible effect on tertiary dentine formation. Therefore, novel 2% QAS when used in combination with 6% NaOCl can be used as an effective antimicrobial irrigant for root canal infections. Further studies are required to ascertain the effect of dentinal fluid exudate and cytoplasmic elongation within dentinal tubules as they may reduce the inward transdentinal diffusion of QAS toward the dental pulp *in vivo*. Preliminary evidence suggested that 2% QAS could outperform CaOH and CHX in certain aspects. Firstly, k21 does not form insoluble precipitates and discoloration, an occurrence commonly found with CHX medicament (Vivacqua-Gomes et al., 2002). Moreover, k21 antibacterial efficacy against *E. faecalis* was at least on par with CHX, while calcium hydroxide was ineffective at killing *E. faecalis*, owing to antimicrobial resistance and poor infiltration of CaOH into dentinal tubules (Louwakul et al., 2017).

QAS had also been used to develop a novel sustained drug delivery system by loading metronidazole onto a quaternary ammonium silane-grafted hollow mesoporous silica (MDZ@QHMS) (Bai et al., 2019). This MDZ@QHMS was very effective with varying efficacies against *S. aureus* and *E. coli*. and the *P. gingivalis* biofilm. A higher concentration of the agent was needed when used against the *P. gingivalis* biofilm, with 150 and 300 μg/ml required to attain minimum inhibitory concentration (MIC) and minimum bactericidal concentration (MBC), respectively (Bai et al., 2019). This could be related to the difference in susceptibility to the contact-killing effect of SiQAC between gram-positive and gram-negative bacteria (Jiao et al., 2017). Metronidazole is probably the component responsible for the antibacterial efficacy against the *P. gingivalis biofilm*. In light of the concern posed by antibiotic resistance, future studies could look into the incorporation of antimicrobial peptides into the QHMS.

In another study, submicron PLGA particles loaded with k21, calcium, and phosphorus elements (P-CaPK) were synthesized. The formation of crystallites induced by P-CaPK was consistent with that of hydroxyapatite, which may assist in occluding dentinal tubules and retarding bacterial ingress. The osteogenic potential could be a product of the silicon, calcium, and phosphorus elements included in this antibacterial system, whereby the silicon acts as nucleation substrate for calcium and phosphorus during mineralization (Damen and Ten Cate, 1989). Inclusion of k21 converted the zeta potential to positive values, which promotes interaction with the negatively charged dentine surface and bacterial cell membranes (Weerkamp and Uyen, 1988). These factors contributed to better dispersion, infiltration, and substantivity of P-CaPK on dentine. This biodegradable drug delivery system (nano-PLGA⋮RF/VE-TPGS0.50 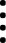 k21) (Daood et al., 2021a,b) was able to reduce biofilm mass, minimize exopolysaccharide production, and aid in the biofilm separation from tooth surfaces. However, differences in dentine histology, fluctuation in pulpal pressure, and the types of adhesive system used are some confounders that can affect nanoparticle infiltration (Mjör and Nordahl, 1996; Berggreen et al., 2007). Their effects on the clinical performance of nano-PLGA⋮RF/VE-TPGS0.50 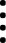 k21 should be ascertained before we can arrive at a definitive conclusion.

Although the results were very promising, they do not reflect the true clinical situation because *in vivo* plaque biofilms are more complex, multi-species, and variegated compared to the mono or dual species biofilms used in most *in vitro* studies. Modern molecular techniques have identified about 1,000 different bacterial species in the dental biofilm, two times as many as can be cultured (Saini et al., 2011). Therefore, further studies are required to validate these preliminary findings by studying its effect on the multi-complex biofilm, as well as conducting more *in vivo* experiments on animal models or even testing its efficacy in clinical trials. Besides clinical efficacy, another factor that should be taken into consideration is its biocompatibility, which will be reviewed in the next section. The primary findings of the major research studies mentioned above are summarized in Table 1.

**Table 1:** Studies showing research on antibacterial potential of QAS as restorative material, cavity disinfectant, root canal medicament, nano-drug delivery.

**Author and year**	**Objective of study**	**Antimicrobial agents used in study**	**Concentration of agents used in study**	**Micro-organism(s) used in study**	**Evaluation method/technique used for testing the antimicrobial properties**	**Specimens used for test**	**Outcome/** **effects** ** (main results (group** ** showing significantly** ** higher bacterial reduction)**	**References**
**Restorative material and cavity disinfectant**								
Gong et al. (2012)	To investigate the antimicrobial activities and nanodynamic mechanical features of a polymerized resin blend of QAMS-3 and bis-GMA.	trifunctional quaternary ammonium methacryloxy siloxanes (QAMS-3) containing SiQAC, TEOS and 3-MPTS.	Bis-GMA: TEGDMA: QAMS-3_PH_ mass ratio of 70:30:0, 70:20:10, 70:10:20, 70:5:25, 70:0:30	*S. mutans, A. naeslundiiand C. albicans*	BacLight LIVE/DEAD viability kit, Confocal laser scan microscopy (CLSM)	Biofilm containing resin disk	Increasing proportion of QAMS-3_PH_ in the polymerized resin is associated with significantly greater antibacterial and antifungal properties (*p* < 0.05), especially for the biofilm cell mass in contact with the resin surface, which was mainly nonviable.	Gong et al., 2012
Zhang et al. (2014)	To evaluate the antibacterial property and dentine bond strength of an experimental antibacterial universal adhesive.	One pot sol-gel synthesized dimethyldiethoxysilane (DMDES) – derived QAMS mixture	7% wt of DMDES-derived QAMS	*S. mutans*	CLSM imaging after staining with LIVE/DEAD BacLight Bacterial Viability Kit; CFU counts; XTT cell metabolism assay	Adhesive coated dentine disk	The experimental universal adhesive with DMDES-derived QAMS demonstrated significantly lower CFU counts and bacteria metabolic activity when compared to the control (*p* < 0.05) without a reduction in microtensile bond strength.	Zhang et al., 2014
Gou et al. (2018)	To assess the inhibitory effect against bacteria embedded in human dentine blocks and the gelatinolytic activity at the resin-dentine interface following application of 2% QAS containing cavity cleanser.	QAS derived from 3-(triethoxysilyl)-propyldimethyloctadecyl ammonium chloride and TEOS	2% QAS cavity cleanser	*S. mutans and A. naeslundii*	CLSM imaging following staining with LIVE/DEAD BacLight Bacterial Viability Kit; CFU counts using sonification method	Dentine blocks	Dentine blocks treated with 2% QAS and 2% CHX had significantly lower CFU counts and higher ratio of dead and live bacteria compared to the control group (*P* < 0.05). The difference in antibacterial action between QAS and CHX is not statistically significant (*P* > 0.05).	Gou et al., 2018
Daood et al. (2019)	To compare the antibacterial activity of different concentrations of QAS cavity cleansers against single and multi-species cariogenic biofilm	QAS cavity cleanser synthesized from TEOS and ethoxy version of SiQAC, CHX	2, 5, and 10% QAS; 2% CHX	*S. mutans, L. acidophilus, A. naeslundii and Streptococcus sanguis*	CSLM; CFU count; MTT assay and Raman analysis	Dentine disks	The proportion of live bacteria in both single and dual-species biofilms of *S. mutans* and *L. acidophilus* decreased significantly with increasing concentration of QAS (*P* < 0.05), with the highest dead cell counts observed for the 10% QAS group.	Daood et al., 2020a
Daood et al. (2020)	To investigate the antimicrobial effects of quaternary ammonium silane (QAS) on *Streptococcus mutans* and *Lactobacillus acidophilus* bacterial biofilms	QAS and CHX	2% CHX, 1% QAS, and 2% QAS	*S. mutans and L. acidophilus*	SEM and DNA-binding 4′,6-diamidino-2-phenylindole (DAPI) analysis, CLSM, time kill assay, fatty acid extraction and succinic dehydrogenase assay, micro-Raman spectroscopy and Sortase A activity inhibition assay	Dentine disks	1 and 2% QAS showed low carbohydrate intensities on Raman spectroscopy. SEM images showed absence of bacterial colonies after treatment. DAPI staining with 1% QAS (*p* < 0.05) was low. 1 and 2% QAS specimens had an increase in the fatty acid compositions of dual species biofilm (*p* < 0.05).	Daood et al., 2020b
**Root canal medication**								
Daood et al. (2019)	To analyze the effect of 2% quaternary ammonium silane (QAS + Sodium hypochlorite (NaOCl) containing novel irrigant against bacteria impregnated inside the root canal system, and its mechanical and antimicrobial potential of dentine substrate.	QAS, Chlorhexidine (CHX) and NaOCl.	6% NaOCl, 2% CHX, and 2% QAS	*E.faecalis*	Confocal laser scan microscopy (CLSM) Scanning electron microscope (SEM), and Raman spectroscopy.	Non-carious human single rooted anterior teeth	2% QAS showed an increased bacterial efficacy when combined with NaOCl as an irrigant impregnated inside a root canal as compared to control and NaOCl + CHX.	Daood et al., 2019
Daood et al. (2020)	To understand the antimicrobial potency, cytotoxicity and mechanical properties of exposed dentine substrate of a novel quaternary ammonium silane irrigant solution (QIS).	QIS, NaOCl, NaOCl + CHX	2 and 3.5% QIS; 6% NaOCl; 6%NaOCl + 2% CHX	*E. faecalis*	LIVE/DEAD BacLight bacterial viability analysis using CLSM; CFU counts; log CFU; Raman spectroscopy	Root canal dentine	*In-vitro* root canal irrigation with QIS significantly reduced the growth of *E. faecalis* biofilm, with the higher concentration of 3.5% QIS effectively eliminating most of the cells. Similarly, average values for CFU/ml were lowest for the 3.5% QIS group. Moreover, structural changes in the biofilm were detected following QIS irrigation, with noticeable reduction in protein, pyocyanin and carbohydrate contents.	Daood et al., 2020c
Kok et al. (2021)	To compare the antimicrobial efficacy of k21 intracanal medicament against *E. faecalis* and *C. albicans* biofilms.	QAS (k21); CHX; CaOH	2% k21; 2% CHX; 41% CaOH	*E.faecalis; C. albicans*	CLSM following BacLight bacterial viability staining; CFU counts; adherence assay; Raman spectroscopy;	Dentine blocks and disks from tooth root	*E. faecalis* and *C. albicans* biofilm decreased significantly (*P* < 0.05) following a change of intracanal medicament from 2% CHX to 2% k21. A lower and high Raman intensities for glycosidic bond and beta-galactosidase enzyme, respectively, suggested a greater destruction of *E. faecalis* by 2% k21.	Kok et al., 2021
**Nano-drug delivery**								
Bai et al. (2019)	To evaluate the antibacterial activity of sustained delivery system of quaternary ammonium silane-grafted hollow mesoporous silica (QHMS) loaded with to metronidazole (MDZ) (MDZ@QHMS) using single-species biofilms.	QHMS, MDZ	MDZ@QHMS (800, 600, 400, 300, 200, 150, 100, 75, 50 and 25 μg/mL)	*S. aureus (ATCC25923), E.coli (ATCC25922) and P. gingivalis (ATCC33277)*.	Colony forming units (CFU), XIT assay, and live/dead bacterial staining.	Bacteria	MDZ@QHMS demonstrated sustained drug release and bacteridalactvity against the three bacterial strains at a concentration of 100 lg/mL or above.	Bai et al., 2019
Fan et al. (2020)	To assess release profiles, antibacterial ability of submicron Poly (D, L-lactic-co-glycolide) [PLGA particles loaded with quaternary ammonium silane (K 21)], calcium and phosphorus against *E. faecalis*, biocompatibility, amount of infiltration into dentinal tubules, and *in vitro* potential of mineralization of particles.	PLGA, submicron PLGA particles containing K21 (P-K), PLGA submicron particles containing calcium and phosphorus (P-CaP), PLGA submicron particles containing K21, calcium and Phosphorus (P-CaPK), and CHX.	2% CHX, 1 mg/ml, 2 mg/ml, 5 mg/ml of P-K, P-CaP and P-CaPK	*E.faecalis*	CFU	Dentine slices	All concentrations of P-K and P-CaPK exhibited potent antibacterial activity against *E. faecalis*. At the same concentration P-CaPK showed stronger bactericidal ability than P-K. Slight antimicrobial activity was seen in the P and P-CaP groups at 2 and 5 mg/mL. Complete inhibition of growth of *E. faecalis* was seen in the 2% CHX positive control (*p* < 0.05).	Fan et al., 2020
Daood et al. (2021)	To analyze the antimicrobial resistance, mechanical properties structural integrity, chemical interactions, of novel k21 PLGA nanoparticles with synergistic effect of d-alpha-tocopheryl poly (ethyleneglycol)-1000-succinate (VE-TPGS) with riboflavin-5-phospshate (RF) on acid-demineralized dentine-substrates.	Blank PLGA, Nano- PLGA⋮RF/VE-TPGS, k21	1, 2, and 3% k21	*S. mutans (ATCC UA159)*	Agar diffusion assay, Biofilm quantification assay, Exopolysaccharides (EPS) quantification assay, Biofilm detachment assay, SEM, CLSM, and Raman spectroscopy.	Demineralized dentine disc	Agar diffusion assay, Biofilm quantification assay, EPS quantification assay, Biofilm detachment assay was best in the 3% k21 group. 3% k21 showed the biofilm disintegration, non-viable cells and the greatest eradicating effect on SEM and CLSM analysis. On Raman spectroscopy, peak intensities were lower in 3 and 1% PLGA groups.	Daood et al., 2021a

## Cytotoxicity of Quaternary Ammonium Silane

Assessing the cytotoxicity of any biomaterial is vital in order for it to be used clinically prior to recommendation for clinical applications. Although cell culture models possess limitations, they still provide crucial data on the effect of biomaterial on cell viability before its use *in vivo*. QAS has been used on cell culture to analyze its *in vitro* effects on dentistry. QAS has exhibited skin reactions and contact dermatitis (Ruiz Oropeza et al., 2011; Iwata et al., 2015). Li et al. (2016) observed that the QAS copolymers, PMT-5%, and PMT-10% were less cytotoxic to three human cell lines compared to QAS monomeric agent DTPAC dimethyloctadecyl[3-(trimethoxysilyl) propyl] ammonium chloride (DTPAC). Moreover, cell viability was found to be relatively high at concentration of 62.5 μg ml^−1^ at which QAS polymer also displayed MIC and MBC for *E. coli* and *S. aureus* (Li et al., 2016). Daood et al. (2017) also showed that the MTT assay depicted higher cell viability of human dental pulp cells (hDPCs) with 2% QAS in comparison with 2% CHX. Cell viability in order of lowest to highest was 19.3–2% CHX, 55.1–2% QAS, and 80.2%- deionized water. Another research by Daood et al. in 2019 showed that 2% QAS exhibited more cell viability with mouse fibroblasts compared to 2% CHX, 5% QAS, and 10% QAS. Thus, an increase in the concentration of QAS is directly proportional to decreased cell viability (Daood and Yiu, 2019). Fan et al. (2020) observed that QAS was very slightly cytotoxic and had superior cell viability compared with 2% CHX. Recent research has shown that QAS with 0.5% and 1% concentration were found to have higher cell viability compared to 2% CHX, 6% NaOCl, 6% NaOCl + 2% CHX (Daood et al., 2021b). Thus, based on research, it is imperative to understand that toxicity is dependent on concentration. Increase in concentration of QAS will also lead to higher toxic effects on the cells, although there needs to be a balance in maintaining the concentration-dependent antibacterial action at (MIC) and MBC with maintenance of biocompatibility of QAS solutions. Please review the outline of all the research studies on cytotoxicity as described in Table 2.

**Table 2:** Research data on cytotoxicity of quaternary ammonium silane.

**Author/year**	**Assessment test use to assess cytotoxicity**	**Cells used for test**	**Biomaterials used for test**	**Outcome of cytotoxicity**	**References**
Li et al. (2015)	MTT assay	Human embryonic kidney 293 (HEK293) cells, human adult low calcium high temperature (HaCaT) keratinocytes human dermal fibroblasts (HDF)	QAS copolymeric agents: PMT-5% and 10%, QAS monomeric agent: DTPAC	QAS copolymeric agent showed lower toxicity compared to QAS monomeric agent	Li et al., 2016
Daood et al. (2017)	MTT assay	hDPCs	Deionised water (control); CHX (2%) QAS (2%, 5%, 10%) concentrations	2% QAS showed more cell viability compared to 2%CHX and deionised water.	Daood et al., 2017
Daood et al. (2019)	Trypan blue assay	NIH 3T3 mouse fibroblasts	deionised water (control); CHX (2%) QAS (2, 5, 10%) concentrations	2% QAS depicted higher ell viability compared to other test solutions	Daood and Yiu, 2019
Fan et al. (2020)	Cell counting kit-8 (CCK-8; Dojindo Laboratories, Kumamoto, Japan)	MC3T3-E1 cells	P, P-CaP, PeK and P-CaPK	PLGA submicron particles with QAS exhibited enhanced biocompatibility compared to 2% CHX.	Fan et al., 2020
Daood et al. (2021)	MTT assay	hDPCs	6% NaOCl, 6%NaOCl + 2%CHX, 2%CHX, 1%k21-E, 0.5%k21-E	K21-E was found to have highest cell viability compared to other test solutions and control	Daood et al., 2021a,b

## Future Prospects

Periodontal disease is associated with a polymicrobial infection. Although implicated in disease, the Red Complex bacteria are present in the microbiome of subjects with clinically healthy gingiva, albeit at lower abundance (Valm, 2019). Thus, the actual microbial communities that are responsible for the transition from periodontal health to a state of dysbiosis are more complex and diverse. Considering polymicrobial dysbiosis in the pathogenesis of periodontal disease, QAS as an antimicrobial agent in the form of chips, gels, fibers, nanospheres should be formulated and tested in the treatment of periodontal diseases. QAS-based mouthwashes, dentifrices could be prospects of daily care products that can be used for routine biofilm control. The osseous defects formed due to periodontal disease need to be regenerated in the three-dimensional planes for successful bone augmentation. A recent study done on novel 3D collagen scaffolds impregnated with novel QAS antibacterial biomaterial, cross-linked with VE-TPGS, has shown that it will provide a unique stable structure in bone formation for osseous defects around teeth and implant. From the clinical viewpoint, it is anticipated that, in the near future, customized 3D scaffolds can be fabricated as per the anatomy of the osseous defects that will ensure complete coverage of the defects with these antimicrobial scaffolds enhancing bone regeneration. Manufacturing of customized 3D collagen scaffolds as per the architecture of osseous defects is considered an innovation in clinical dentistry for increasing the longevity of teeth (Bapat et al., 2021). *In vitro* research should be performed to investigate SrtA inhibitory activity of QAS. Lower SrtA half maximal inhibitory concentration (IC_50_) values along with higher MIC value will serve as an indicator of QAS's potential to interrupt action of bacteria, maintaining its viability without drug resistance. Therefore, future studies should look into harnessing the antibacterial and anti-inflammatory properties of QAS in the treatment of periodontal diseases.

## Conclusion

This review has demonstrated QAS's antimicrobial activities against *E. faecalis, S. mutans*, and *L. acidophilus* that can lead to successful treatment outcomes in clinical dentistry. The compound enables contact killing of the bacteria, leading to cell lysis. QAS also demonstrated biocompatibility with mammalian cells, suggesting its application as a novel dental biomaterial with minimal risk to dental health.

## Author contributions

UD: conceptualization, funding acquisition, and supervision. RB: data curation. UD and RB: formal analysis. RB, UD, AP, and PK: investigation. RB, AP, and PK: methodology. SL: project administration. SL, TC, and HY: resources. RB and TC: software. SL and KP: validation. RB and HY: roles/writing—original draft. UD, SL, and KP: writing—review and editing. All authors contributed to the article and approved the submitted version.

## Funding

The study was supported by KHG fiteBac Technology, Marietta, GA, USA. The study protocol was approved by the Institutional Research Ethical Committee 259/2020 and FRGS/1/2020/SKK0/IMU/02/10 project.

## Conflict of Interest

The authors declare that the research was conducted in the absence of any commercial or financial relationships that could be construed as a potential conflict of interest.

## Publisher's Note

All claims expressed in this article are solely those of the authors and do not necessarily represent those of their affiliated organizations, or those of the publisher, the editors and the reviewers. Any product that may be evaluated in this article, or claim that may be made by its manufacturer, is not guaranteed or endorsed by the publisher.

## Abbreviations

CHX: Chlorhexidine
CPC: Cetylpyridinium chloride
MIC: Minimum inhibitory concentration
*S. mutans*: *Streptococcus mutans*
*P. intermedia*: *Prevotella Intermedia*
*S. sobrinus*: *Streptococcus sobrinus*
*S. sobrinus*: *Streptococcus sobrinus*
NaOCl: sodium hypochlorite
SiQAC: 3-(trimethoxysilyl)-propyldimethyloctadecyl ammonium chloride
QAC: quaternary ammonium compounds
QAS: Quaternary ammonium silane
TEOS: tetraethoxysilane
QAS/k21: quaternary ammonium silanes
SrtA: Sortase A
*A. naeslundii*: *Actinomyces naeslundii*
*C. albicans*: *Candida albicans*
CLSM: confocal laser scanning microscopy
CFU: Colony forming unit
XTT: Cell Proliferation Kit
SrtA: Sortase A
MMP: Matrix metalloproteinases
SEM: Scanning Electron Microscopy
SEM: Scanning electron microscopy
*E. faecalis*: *Enterococcus faecalis*
*E. coli*: *Escherichia coli*
P-CaPK: calcium and phosphorous
QAMS-3: trifunctional quaternary ammonium methacryloxy siloxanes
3-MPTS: 3-methacryloxypropyltrimethoxysilane
DMDES: dimethyldiethoxysilane
NaOCl: Sodium hypochlorite
DTPAC: dimethyl octadecyl [3-(trimethoxysilyl) propyl] ammonium chloride
hDPCs: human dental pulp cells.
